# Dentition and Diarrhœa

**Published:** 1897-08

**Authors:** F. Paschal

**Affiliations:** San Antonio, Texas


For the Texas Medical Journal.

### DENTITION AND DIARRHŒA.

BY F. PASCHAL, M. D., SAN ANTONIO, TEXAS.

[Read at meeting of West Texas District Medical Society, San Antonio, Texas.]

THERE is a prevalent belief among the laity, and especially the women part of it, and which is shared by some of the members of our profession, that many of the gastro-intestinal disorders occurring during dentition are physiological, and therefore to be expected. That gastro-intestinal disorders do occur so frequently at the time of teething as to make this belief accepted by many, we well know, but that gastro-intestinal disorders are caused by dentition I do not believe can be substantiated. It has been my experience that properly fed infants are, as a rule, constipated instead of having diarrhœa during the first two years of their lives. It is true that the gastro-intestinal tract is undergoing rapid changes at the time of dentition, and is more easily deranged than it is after dentition, but the underlying cause for these derangements is not the physiological processes, but others, chief among which is improper feeding. It may seem to many of you that this is an unimportant subject to bring before you this hot night, but the advent of the hot weather is a reminder of the fact that the greatest mortality among infants is during just such weather. The mortality of infancy is variously estimated at from 15 to 30 per cent. during the first year of life. Within a few years returns from Berlin, French and Italian cities, and even Boston placed the mortality of infants of one year or less at from 20 to 30 per cent. Nor are these statistics exaggerated by the inclusion of still births. Of course there are many diseases of infancy responsible for this large mortality, but nevertheless careful compilations estimate that 42 per cent. of deaths occurring amongst infants are due to digestial disorders. It is a notable fact that in Norway where almost every child is nursed, it is only 10 per cent. In Wurtemburg the mortality of breast-fed children is 13.5 per cent., and of artificially fed children it is 42.7. In saying that breast milk is the proper food for infants during the first year of their life is stating a too well known fact, but unfortunately there are more mothers who do not have enough nourishment for their infants than those who do have enough. Of 525 cases analyzed at the Freisburg maternity only one-half were able to nurse their babies during the first two weeks. In 99 no milk was secreted; in 46 imperfect nipples; in 46 fissures; in 44 insufficient secretion. Only 33 suckled freely without complications.

With this large percentage of mothers unable to nurse their children, it is certainly desirable to find some substitute for infants deprived of the breast; and if I were to bore you with the number of proposed substitutes, I am sure you would never invite me to read another paper. The most available one is cow's milk, but this is subject to so many conditions as to make it an element of importance to every health department, and unless proper measures are adopted it is a danger to every community. Many homicides are probably committed, traceable to infected milk, and which are preventable. There is no better media for germ life than cow's milk, and barring the fact that an infant's stomach is often made a truck farm of where anything from fruits and vegetables to bacon is forced into it, accepting the belief that various contagious germs, as typhoid fever, scarlet fever, diphtheria and tuberculosis, have been found in milk, and no doubt have been the cause of death. Yet these are insignificant factors of danger as compared to the bacteria with which milk is found to be swarming after an exposure of two hours in such weather as we are now having, and it is here that the chief cause of digestial disorders is to be found during dentition; and here, also, is an explanation of such a large mortality during the first year of infant life, whether from that rapid toxæmia causing the so-called "cholera infantum," and which we know is nothing more or less than a ptomaine poisoning, or whether from a less rapid intestinal septicæmia, or from deeper involvement, causing gastro-intestinal inflammation and ulceration. However the case may end, in the very great majority of cases it is from infected food, of which milk forms the principal part. We eat cooked foods and are less liable to being poisoned; the infant depends upon what it gets—mostly uncooked milk.

As we must rely upon condensed milk as a food for infants we should, after prescribing it in such proportions as the age and conditions of the infants require, order that it be boiled and put in sterilized bottles tightly corked, and kept on ice in a cool place. Of course, where one has an intelligent family to deal with, more elaborate means, or possibly better means can be adopted, but as a rule some easy means must be suggested for sterilizing or nothing will be done. I think it best to order it put in bottles well stopped, for if kept in an ice chest uncovered or exposed to the air, it only becomes infected less slowly, but infected all the same. A glance into an ice chest will convince one that these valuable repositories for meats, vegetables, cooked and raw fruits, butter, eggs—smeared over with one broken a few days before—and snugly placed among the promiscuous mass is baby's milk, getting the full benefit of a mixed infection of animal and vegetable germs; and poor baby, if taken ill with diarrhœa during teething, why, of course, it is its teeth. The old ladies think so, and of course, many doctors think with the old ladies—that it is the child's teeth, and if baby dies, why, it died of teething.

Now one word about artificial food and I am through. For my part I have no faith in them, and I think that what cannot be accomplished with condensed milk, modified to meet requirements, will not be accomplished by the many nostrums in the food line, prepared to fill the coffers of the manufacturers. I do not think it is beneath us to educate people to the facts which recent science and perfect experimentation, aided by that most of all valued instruments, the microscope, have taught us; for every human life saved, however insignificant it may be, even the life of an infant, certainly reflects the highest gift of our profession, for after all we are merely tools for the purpose of benefitting our race.

